# Pharmacokinetic Compatibility of Ginsenosides and *Schisandra* Lignans in *Shengmai-san*: From the Perspective of P-Glycoprotein

**DOI:** 10.1371/journal.pone.0098717

**Published:** 2014-06-12

**Authors:** Yan Liang, Yuanyuan Zhou, Jingwei Zhang, Tai Rao, Lijun Zhou, Rong Xing, Qian Wang, Hanxu Fu, Kun Hao, Lin Xie, Guangji Wang

**Affiliations:** Key Lab of Drug Metabolism & Pharmacokinetics, China Pharmaceutical University, Nanjing, Jiangsu Province, China; Taipei Medical University, Taiwan

## Abstract

**Background:**

Phytochemical-mediated alterations in P-glycoprotein (P-gp) activity may result in herb-drug interactions by altering drug pharmacokinetics. *Shengmai-san*, a traditional Chinese herbal medicine composed by *Panax Ginseng, Ophiopogon Japonicus, and Schisandra Chinensis*, is routinely being used for treating various coronary heart diseases. In our previous studies, S*chisandra Lignans Extract* (SLE) was proved as a strong P-gp inhibitor, and herein, the compatibility of *Shengmai-san* was studied by investigating the influence of SLE on the pharmacokinetics of the ginsenosides from the perspective of P-gp.

**Methodology:**

Pharmacokinetic experiments were firstly performed based on *in vitro* uptake, efflux and transport experiments in Caco-2, LLC-PK1 wild-type and MDR1-overexpressing L-MDR1 cells. During the whole experiment, digoxin, a classical P-gp substrate, was used as a positive control drug to verify the cells used are the valid models. Meanwhile, the effects of SLE on the pharmacokinetics of ginsenosides were further investigated in rats after single-dose and multi-dose of SLE.

**Results and Conclusions:**

The efflux ratios of ginsenoside Rb2, Rc, Rg2, Rg3, Rd and Rb1 were found more than 3.5 in L-MDR1 cells and can be decreased significantly by verapamil (a classical P-gp inhibitor). Contrarily, the efflux ratios of other ginsenosides (Rh1, F1, Re, and Rg1) were lower than 2.0 and not affected by verapamil. Then, the effects of SLE on the uptake and transport of ginsenosides were investigated, and SLE was found can significantly enhance the uptake and inhibit the efflux ratio of ginsenoside Rb2, Rc, Rg2, Rg3, Rd and Rb1 in Caco-2 and L-MDR1 cells. Besides, *In vivo* experiments showed that single-dose and multi-dose of SLE at 500 mg/kg could increase the area under the plasma concentration time curve of Rb2, Rc and Rd significantly without affecting terminal elimination half-time. In conclusion, SLE could enhance the exposure of ginsenosides Rb2, Rc, Rg2, Rg3, Rd and Rb1 significantly.

## Introduction

Traditional Chinese medicine (TCM) has been utilized in the Chinese culture to treat the complex of symptoms for thousands of years [1], [2]. As the comprehensive effect of medical system, TCM advocates combinatory therapeutic strategies by prescriptions called ‘formulae’ which can hit multiple targets and produce a synergistic therapeutic effect based on their multi-targeting, multi-ingredient preparations, in contrast to modern pharmacology and drug development that often focus on a single chemical entity [3], [4]. In TCM theory, compatibility refers to the combined use of two or more herbal medicines based on the clinical efficacy and the properties of each herb, and the principles of herbal medicine in TCM can be divided into “monarch”, “minister”, “assistant” and “messenger” [5], [6]. Among them, the “monarch” drug directly aims at the main disease or principal syndrome, and “minister” drug has auxiliary therapeutic effect on the main or accompanying syndrome, as well as to aim at the accompanying diseases or symptoms. Besides, “assistant” drug is used to promote “minister” drug treat the chief syndromes / disease, or to aim at minor symptoms, and the “messenger” drug mainly used to modulate the interactions among the herbs [7], [8]. TCM compatibility has shown its great significance in long-term clinical practices, and revealing the compatibility mechanism of TCM is a key issue to its modernization, while the research has faced many obstacles due to the unimaginable complexity of multi-herb formulae [9], [10]. In fact, pharmacokinetic interactions among the bioactive constituents of herbal formulae can supply important clues to the compatibility research for TCM. In recently years, there was an increasing focus on the compatibility study for TCM based on pharmacokinetic interaction among herbs [11]–[13].

*Shengmai-san*, composed by *Panax Ginseng, Ophiopogon Japonicus, and Schisandra Chinensis*, has been applied for cardiovascular diseases routinely and prophylactically for thousands of years in China as a well-known traditional Chinese herbal prescription [14]–[16]. Among them, *Panax Ginseng* was monarch drug, *Ophiopogon Japonicus* was minister drug, and *Schisandra Chinensis* acted as the role of assistant and messenger drug. According to previous reports, several kinds of *Schisandra* lignans were found can alter pharmacokinetics of substrates of P-gp [17]–[19]. In recent years, several publications have already discussed the relationship between ginsenosides and P-gp [20]–[25], and most of the studies are carried based on the single component of *Ginsenosides Extract*. However, the curative effects of traditional Chinese medicines are principally based on the synergic effect of their multi-targeting, multi-ingredient preparations, in contrast to modern pharmacology and drug development that often focus on a single chemical entity. P-gp, a member of the ATP binding cassette, plays an important role in the exposure and disposition for many xenobiotics through its action as an efflux pump [26]. Thus, phytochemical-mediated alterations in P-gp activity may result in herb-drug interaction by altering drug pharmacokinetics [27]. In 2013, SLE was proved as a strong P-gp inhibitor, and could significantly down-regulate of the P-gp expression and inhibit P-gp activity by our research group [28]. *Schisandra Chinensis*, an assistant and messenger drug in *Shengmai-san*, was used to improve the pharmacological activity of monarch drug (*Panax Ginseng*) and ministerial drug (*Ophiopogon Japonicus*). However, the mechanism of improving the pharmacological activity produced by *Schisandra Chinensis* in *Shengmai-san* was not clear until now. Herein, the compatibility of *Shengmai-san* was studied by investigating the influence of *Schisandra lignans* on the pharmacokinetics of the ginsenosides from the perspective of P-gp.

In this study, several kinds of ginsenosides were systematically proved as P-gp substrates through investigating the effects of P-gp inhibitor (verapamil) on ginsenosides transport and uptake on in Caco-2, LLC-PK1 wild-type and MDR1-overexpressing L-MDR1 cells. Digoxin, a classical P-gp substrate, was used as a positive control drug to verify the cells used are the valid models. Firstly, ginsenoside Rb2, Rc, Rg2, Rg3, Rd and Rb1 were confirmed as P-gp substrates. Then, the effects of SLE on the pharmacokinetics of ginsenoside Rb2, Rc, Rg2, Rg3, Rd and Rb1 were thoroughly investigated based on *in vitro* and *in vivo* models, and SLE was found can greatly enhance the exposure of ginsenoside Rb2, Rc, Rg2, Rg3, Rd and Rb1 in cells and rats. According to the results above, the profound mystery of compatibility principles for *Shengmai-san* may have been revealed to some extent.

## Materials and Methods

### Animals

This study was carried out in strict accordance with the recommendations in the Guide for the Care and Use of Laboratory Animals of the National Institutes of Health. The protocol was approved by the Committee on the Ethics of Animal Experiments of the China Pharmaceutical University. All surgery was performed under sodium pentobarbital anesthesia, and all efforts were made to minimize suffering. Female healthy Sprague-Dawley rats (200±20 g) were purchased from the Laboratory Animal Center of Peking University Health Science Center (Beijing, PR China) and kept in an environmentally controlled breeding room for at least 3 days before experimentation. The rats were fed with standard laboratory food and water and fasted overnight but with free access to water before the test.

### Materials

SLE was purchased from Nanjing Qingze Medical Technological Development Co. Ltd (Nanjing, China). *Ginsenosides Extract* (GE) was purchased from Jilin University (Jilin, China). Digoxin and verapamil were purchased from Sigma-Aldrich (St. Louis, MO). Ginsenoside Rh1, F1, Rb2, Rc, Rg2, Rg3, Re, Rd, Rb1 and Rg1 were purchased from Jilin University (Jilin, China), and their structures are shown in Figure S1. HPLC-grade acetonitrile and methanol were purchased from Merck (Merck, Germany). Deionized water was prepared by the Milli-Q system (Millipore Corporation, Billerica, MA) and was used throughout. Other reagents and solvents were commercially available and of analytical grade.

### Methods

#### LLC-PK1 and L-MDR1 cell culture

LLC-PK1 cells, a cell line lacking P-gp, and the derived cell line L-MDR1, which over-express human MDR1-encoded P-gp [29], were grown in minimum essential medium with supplements at 37°C, 5% CO_2_, and 95% humidity. LLC-PK1 and L-MDR1 cells were seeded at a density of 12×10^6^ cells per 150-cm^2^ flask and grown for 7 days in medium 199 supplemented with 2 mM glutamine, 10% fetal bovine serum, 10 mg/500 mL of streptomycin, and 10,000 IU/500 mL of penicillin. To ensure a constant expression level of transport protein, L-MDR1 cells were grown in the presence of 640 nM vincristine. Cells were grown as monolayer on polycarbonate membrane filters (Transwell; Costar Corporation, Cambridge, MA) as outlined previously [30]. Transepithelial resistance was measured in each well using a Millicell ERS ohmmeter (Millipore, Bedford, MA); wells registering a resistance of 200 ohms or greater, after correcting for the resistance obtained in control blank wells, were used in the transport experiments.

#### Inhibiting transport of ginsenosides by verapamil and SLE in cultured LLC-PK1 and L-MDR1 cells

The LLC-PK1 and L-MDR1 cells were washed with warm Hank's balanced salt solution HBSS (pH 7.4) twice before transport experiment. HBSS containing verapamil (10 µM), SLE (2.0 and 10.0 µg/mL) or blank HBSS (control) was then loaded into both apical and basolateral chambers. After incubation at 37°C for 1.5 h, GE (100 µg/mL) was added to either the apical or basolateral side to evaluate the transport in absorptive and secretive directions, respectively. The cell monolayer was then incubated for another 2 h. Finally, the concentrations of ginsenosides were determined by LC/MS. All experiments were conducted in triplicate.

#### Enhancing uptake of ginsenosides by verapamil and SLE in cultured LLC-PK1 and L-MDR1 cells

LLC-PK1 and L-MDR1 cells were incubated with SLE (2.0 and 10.0 µg/mL) or verapamil (10 µM) for 1.0 h, and then were washed to discard SLE or verapamil before adding 100 µg/mL of GE. The accumulation of GE lasted for 2 h, and the intracellular concentrations of ginsenosides were measured by LC/MS. All experiments were conducted in triplicate.

#### Caco-2 cell culture

Caco-2 cells were purchased from American Type Culture Collection (Manassas, VA), and were routinely cultured in Dulbecco's modified Eagle's medium supplemented with 10% fetal bovine serum, 1% nonessential amino acids, 1 mM sodium pyruvate, and 100 U/mL penicillin and streptomycin (Invitrogen, Carlsbad, CA). The cells were grown in an atmosphere of 5% CO_2_ at 37°C, and cell medium was changed every other day. Cells were seeded at a density of 4×10^4^ cells per well in 24-well microporous polycarbonate insert filter plates (0.4-µm pore size) (Corning Costar plates; Corning Life Sciences, Lowell, MA) and grown for 15 days in Caco-2 growth medium. The medium was replaced every 3 to 4 days. Before running the assay, the culture medium was replaced by HBSS buffer supplemented with 10 mM Hepes and 20 mM glucose.

#### Inhibiting transport of ginsenosides by verapamil and SLE in Caco-2 cell monolayer

Caco-2 cells with a density of 1.2×10^5^ cells/insert were seeded on a permeable polycarbonate insert (Millicell cell culture inserts, 0.4 µm pore size, 12 mm diameter; Millipore Corporation) in 24-well tissue culture plates and were used for the experiment 18 to 21 days after seeding. [^14^C]Mannitol permeability and transepithelial electrical resistance measurements (Millicell-ERS epithelial volt-ohm meter; Millipore Corporation) were used to evaluate the integrity of Caco-2 cell monolayer. The monolayer used in transport studies had transepithelial electrical resistance values exceeding 600 Ω·cm^2^, and the leakage of mannitol was less than 0.3% of the dose/h. Before transport initiation studies, the cell monolayer was first washed with warm HBSS (pH 7.4) twice. HBSS containing SLE (2.0 and 10.0 µg/mL) or 1% ethanol (control) was then loaded into both apical and basolateral chambers. After incubation at 37°C for 1.5 h, 100 µg/mL of GE was added to either the apical or basolateral side to evaluate the transport in absorptive and secretory directions, and the cell monolayer was incubated for another 2 h. 10 µM verapamil was used as a positive control inhibitor. At the designated time point, samples were taken from the receiving chamber for analysis. Ginsenosides were determined by LC-MS. All experiments were conducted in triplicate.

#### Enhancing uptake of ginsenosides by verapamil and SLE in Caco-2 cell

Caco-2 cells were incubated with SLE (2.0 and 10.0 µg/mL) or verapamil (10 µM) for 1.0 h. Then the cells were washed with Hank's to discard SLE or verapamil before the addition of GE (100 µg/mL). The accumulation of ginsenosides lasted for 2 h, and the intracellular concentrations of ginsenosides were measured by LC/MS. All experiments were conducted in triplicate.

#### In vivo pharmacokinetic studies

To evaluate the effect of single treatment of SLE on the pharmacokinetics of ginsenosides, 24 rats were divided into four groups (group I ∼ group IV) averagely. In group I: the rats were administrated a single dose of SLE at 500 mg/kg by gavage. 2 h later, digoxin (0.5 mg/kg), a P-gp substrate, was given to the rats by intragastric administration. In group II (control group): the rats received a single dose of vehicle (0.5% CMC-Na), and digoxin (0.5 mg/kg) was also given to the rats by intragastric administration. In group III: the rats received a single dose of SLE at 500 mg/kg suspended in 0.5% CMC-Na. 2 h later, GE was administrated to the rats at a dose of 120 mg/kg by gavage. In group IV (control group): the rats received a single dose of vehicle (0.5% CMC-Na), and GE (120 mg/kg) was also given to the rats by gavage.

To evaluate the effect of long-term treatment with SLE on the pharmacokinetics of ginsenosides, 24 rats were also divided into four groups (group I∼ group IV) with 6 animals each. In the group I and group III, SLE was administrated intragastrically to rats at a dose of 500 mg/kg once a day for 10 continuous days, whereas the other two groups (group II and group IV) received the vehicle (0.5% CMC-Na), serving as the control groups. On the 11^th^ day, digoxin was administered intragastrically to rats (group I and group II) at a single dose of 0.5 mg/kg, GE was administered intragastrically to rats (group III and group IV) at a single dose of 120 mg/kg.

In the experiments above, 0.2 mL blood samples were collected at 5, 10, 20, 40, 60, 120, 240, 360, 480 and 720 min after digoxin dosing. After intragastric administration of GE, 0.2 mL blood samples were collected at 5, 10, 20, 40, 60 min, 2, 4, 6, 8, 12, 24, 48, 72 and 96 h. Plasma was obtained by centrifugation at 5000 *g* for 10 min and stored at −20°C before analysis.

#### Analysis of ginsenosides in cell Lysates, buffer Samples and rat plasma

The primary stock solutions of 10 ginsenosides were prepared by dissolving 10.0 mg of corresponding standards in 10 mL acetonitrile, and were stored at 4°C until analysis. Working solutions of the analytes were prepared by appropriate dilution of the primary stock solutions in acetonitrile. Then the stock solutions were diluted with blank rat plasma to make the calibration standards at the concentrations of 0.001, 0.002, 0.005, 0.01, 0.02, 0.05, 0.10, 0.20, 0.50, 1.00, 2.00, 5.00 and 10.00 µg/mL for all the ginsenosides.

The plasma / cell samples were extracted using a liquid–liquid extraction technique. To each tube containing 100 µL plasma, 10 µL internal standard (digoxin, 1.0 µg/mL) and 1.0 mL of *n*-butanol were added. The mixture was then vortex-extracted for 2 min, and centrifuged for 10 min at 10,000 g. The *n*-butanol extract was evaporated to dryness in a rotary evaporator and the residue was reconstituted in 200 µL acetonitrile. 5 µL aliquot was analyzed by LC/MS.

LC experiments were conducted on a Shimadzu (Kyoto, Japan) HPLC system. Chromatographic separation was achieved on a Phenomenex C_18_ column (150 mm×2.0 mm, 5 µm, Lunar) at 40°C. Water mobile phase (solvent A) was H_2_O containing 0.1 mM ammonium chloride; and the organic phase (solvent B) was acetonitrile. A 30-minute binary gradient elution (delivered at 0.2 mL/min) was performed for the separation: an isocratic elution of 30 % solvent B for the initial 1.0 min, followed by a linear gradient elution of 30–75% solvent B from 1.0 to 23 min; after holding the composition of 75 % solvent B for the next 2 min, the column was returned to its starting conditions till the end of the gradient program at 30 minute for column equilibration.

The ESI source was used in negative ionization mode. The [M+CL]^−^ ions of ginsenosides (Re, Rg1, Rb1, Rb2, Rg2, Rc, Rh1, F1, Rd, and Rg3 ) and internal standard (digoxin, *m/z* 815.55) were used for SIM detection, the chromatography of ginsenosides are shown in Figure S2. Peak areas were used for quantification. The optimum MS operating conditions were as follows: drying gas 2.5 L min^−1^, curved desolvent line temperature 250°C, block temperature 200°C, detector voltage 1.65 kV, peak width 0.7 FWHM, interval time 0.2 s.

#### Data Analysis

For the transport assay, the apparent permeability (*P*_app_, cm/s) in each direction (A→B and B→A) was calculated as follows, where *A* is the surface area of the membrane inserts (0.0804 cm^2^), *C*_0_ is the initial concentration of the compound applied in the donor compartment (2 µM), and △*Q* is the amount (micromoles) of compound transported over time △*t* (5 h = 18,000 s) [31]: 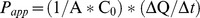


The efflux ratio (ER) was a dimensionless number calculated as the ratio of the apparent permeability in the B→A direction divided by the apparent permeability in the A→B direction. 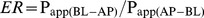



#### Calculation of pharmacokinetic parameters

The plasma concentration-time curves of ginsenosides in rats were obtained by plotting the mean plasma concentrations versus time. Pharmacokinetic parameters were calculated using the WinNonlin 6.3 program (Pharsight Inc., Mountain View, CA). The elimination half-life (*t*_1/2_) value was calculated as 0.693/β, where β is the elimination rate constant calculated from the terminal linear portion of the log plasma concentration-time curve. The total areas under the plasma concentration- time curve from time 0 to the last quantifiable time point (AUC_0-t_) and from time 0 to infinity (AUC_0-∞_) were calculated using the log trapezoidal rule. 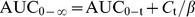



The maximum plasma concentrations (*C*_max_) for ginsenosides were obtained by visual inspection of the plasma concentration-time curve, whereas the initial drug concentration (the extrapolated concentration at zero time) of the drug after intravenous injection was calculated by back-extrapolation of the plasma concentration-time curve to the *y*-axis.

### Systematic workflow

**Figure 1:**
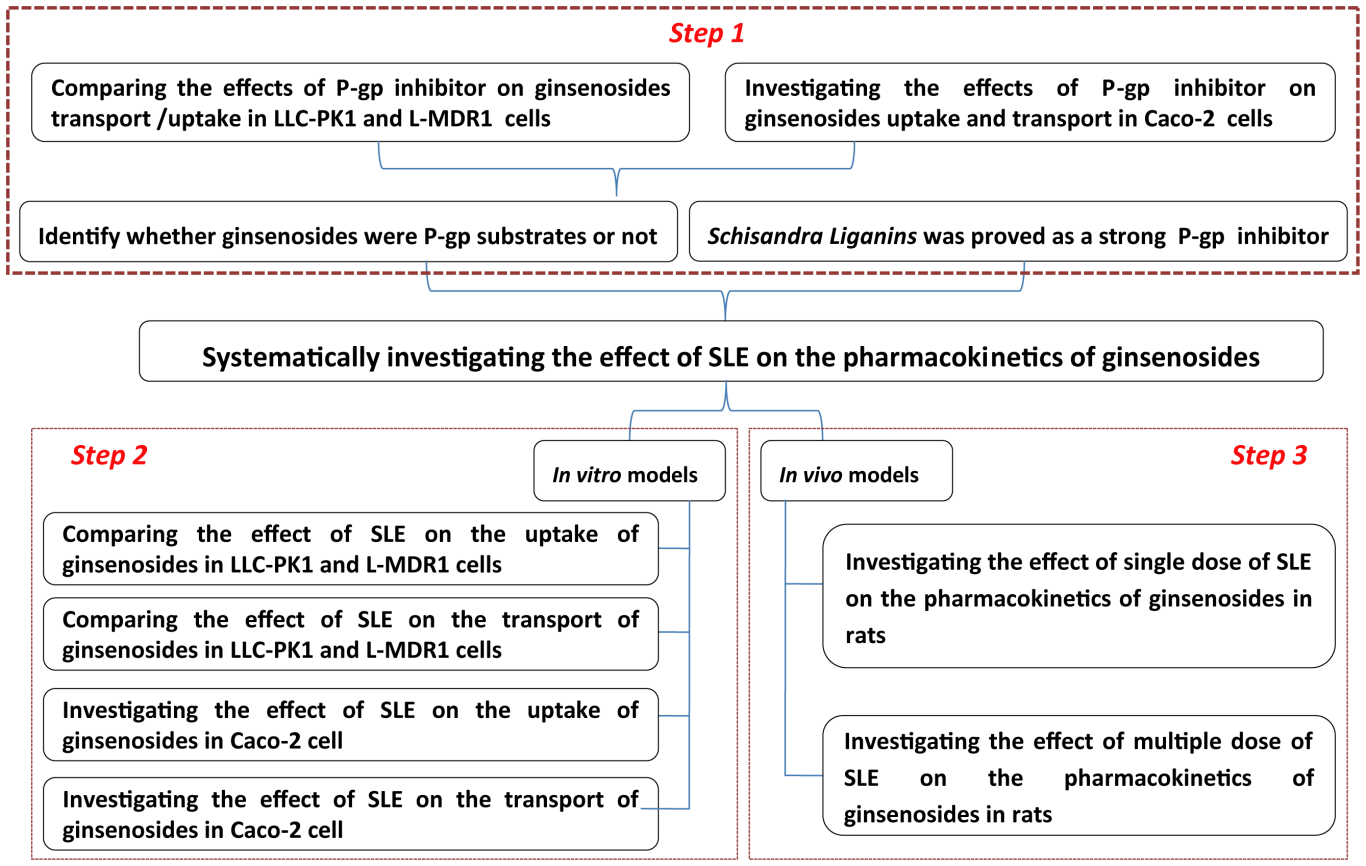
The systematic workflow of this study.

The systematic workflow is schematically depicted in Figure 1. In step 1, ginsenosides were identified as P-gp substrates through comparing the effects of P-gp inhibitor on ginsenosides transport and uptake in LLC-PK1 wild-type and MDR1-overexpressing L-MDR1 cells. Besides, the effects of P-gp inhibitor on transport and uptake in Caco-2 cell were also investigated to further confirm several kinds of ginsenosides were P-gp substrates. In step 2, the effects of SLE on the transport and uptake of ginsenosides were inspected in Caco-2, LLC-PK1 and L-MDR1 cells since SLE were reported as a strong P-gp inhibitor, and in step 3, the effects of SLE on the pharmacokinetics of ginsenosides were investigated *in vivo* through comparing the pharmacokinetics of ginsenosides in rats after single/ multiple dose of SLE with that in the control rats which received the vehicle except GE.

## Results

### The effects of P-gp inhibitor on ginsenosides transport and uptake

In order to identify whether ginsenosides are P-gp substrates or not, we compared the effect of a classical P-gp inhibitor (verapamil) on the transport / uptake abilities of ginsenosides in LLC-PK1 cells and the derived cell line L-MDR1. Besides, the transport and uptake of ginsenosides were also investigated on Caco-2 cell in the absence and presence of verapamil to further confirm which ginsenosides were P-gp subtrates. In order to verify the reliability of the cell models, digoxin was used as a positive control drug during the whole course of the experiment.

#### The effects of P-gp inhibitor (verapamil) on Ginsenosides transport in cultured LLC-PK1 and L-MDR1 cells

**Figure 2:**
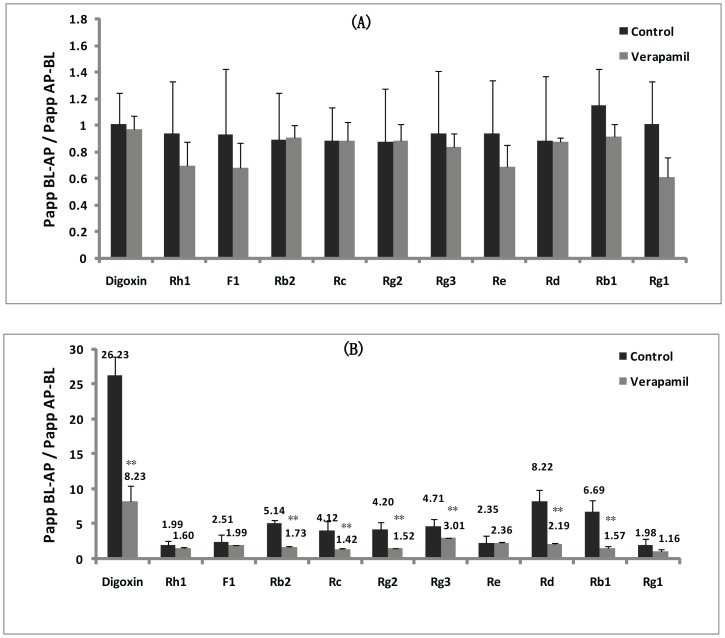
The effect of verapamil on the efflux ratio of digoxin, ginsenoside Rh1, F1, Rb2, Rc, Rg2, Rg3, Re, Rd, Rb1 and Rg1 on LLC-PK1 and L-MDR1 cell. (A) The efflux ratio of digoxin and ginsenosides on LLC-PK1 cells in the absence and presence of verapamil. (B) The efflux ratio of digoxin and ginsenosides on L-MDR1cells in the absence and presence of verapamil.

The effect of verapamil (a classical P-gp inhibitor) on the transport ability of ginsenosides has been investigated in LLC-PK1 cells and the derived cell line L-MDR1. Figure 2(A) illustrates the ERs of digoxin (positive control drug), ginsenoside Rh1, F1, Rb2, Rc, Rg2, Rg3, Re, Rd, Rb1 and Rg1 on LLC-PK1 cells (lacking P-gp), in the absence and presence of 10 µM verapamil as a P-gp inhibitor. No transport difference was observed when verapamil was added, and the efflux level of digoxin and ginsenosides across LLC-PK1 cell monolayer in the absorptive (AP→BL) was similar to the secretive (BL→AP) directions. It was mean that the ER of ginsenosides across LLC-PK1 cell monolayer and the corresponding *P*_app_ values were not affected by verapamil. On the other hand, we also compared the transport of digoxin, ginsenoside Rh1, F1, Rb2, Rc, Rg2, Rg3, Re, Rd, Rb1 and Rg1 on L-MDR1 cells (LLC-PK1 cells stably transfected with the human MDR1 gene), in the absence and presence of 10 µM verapamil as P-gp inhibitor (shown in Figure 2(B)). Obviously, the ERs of digoxin, ginsenoside Rb2, Rc, Rg2, Rg3, Rd and Rb1 on L-MDR1 cells were 26.23, 5.14, 4.12, 4.20, 4.71, 8.22 and 6.69, respectively (far greater than 2.0). Moreover, the addition of verapamil caused a significantly decrease of ER through increasing the transport of digoxin, ginsenoside Rb2, Rc, Rg2, Rg3, Rd and Rb1 in the AP→BL direction, and decreasing their transport across L-MDR1 cell monolayers in the BL→AP direction. For the other ginsenosides (Rh1, F1, Re and Rg1), the ERs across L-MDR1 cells were lower than 2.0, and the corresponding *P*_app_ values were almost not affected by verapamil (*P>0.05*).

#### The effects of verapamil on the uptake of ginsenosides in cultured LLC-PK1 and L-MDR1 cells

**Figure 3:**
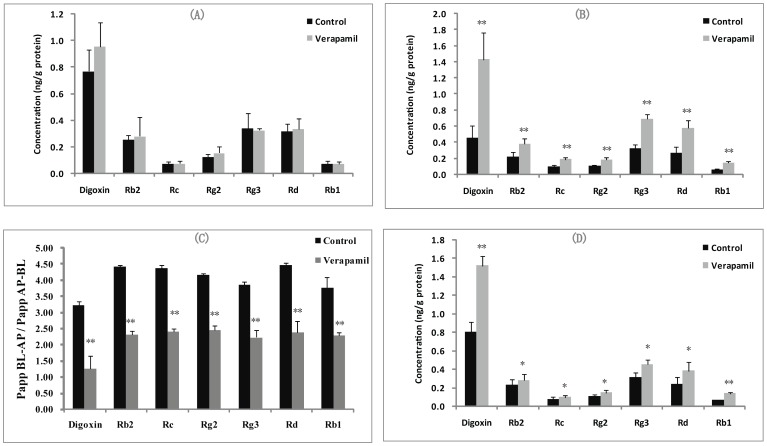
The effect of verapamil on the uptake of ginsenosides in LLC-PK1 and L-MDR1 cell and the effect of verapamil on the uptake and transport of digoxin and ginsenosides in Caco-2 cell. (A) The concentration of digoxin and ginsenosides in LLC-PK1 cell in the absence and presence of verapamil. (B) The concentration of digoxin and ginsenosides in L-MDR1 cell in the absence and presence of verapamil. (C) The concentration of digoxin and ginsenosides in Caco-2 cell in the absence and presence of verapamil. (D) The efflux ratio of digoxin and ginsenosides on Caco-2 cells in the absence and presence of verapamil.

Herein, we examined the effect of verapamil on the uptake of ginsenosides on LLC-PK1 cells and the derived cell line L-MDR1. As shown in Figure 3(A), intracellular accumulation of digoxin, ginsenoside Rb2, Rc, Rg2, Rg3, Rd and Rb1 on LLC-PK1 cells had no significant difference in the absence and presence of verapamil. In contrast, the intracellular concentrations of digoxin, ginsenoside Rb2, Rc, Rg2, Rg3, Rd and Rb1 on L-MDR1 cells were greatly increased in the presence of P-gp inhibitor verapamil (Figure 3(B)), and the uptake levels of ginsenoside Rb2, Rc, Rg2, Rg3, Rd and Rb1 were enhanced by 3.2-, 1.7-, 1.5-, 1.6-, 1.5-, 1.8 and 2.6- fold in the presence of 10 µM verapamil, respectively (*P<0.05*).

#### The effects of verapamil on the transport and uptake of ginsenosides in Caco-2 cell

Caco-2 cells, a human colon adenocarcinoma cell line, had demonstrated numerous morphological and biochemical characteristics of enterocytes with a well-developed brush border and associated enzymes and transporters [32]. Herein, we studied the effect of verapamil on the transport ability of ginsenosides in Caco-2 cell. Figure 3(C) illustrates the ERs of digoxin, ginsenoside Rb2, Rc, Rg2, Rg3, Rd and Rb1in Caco-2 cells, in the absence and presence of 10 µM verapamil as P-gp inhibitor. Ginsenoside Rb2, Rc, Rg2, Rg3, Rd and Rb1 exhibited highly polarized transport across Caco-2 cell monolayers with marked efflux ratio value at 3.23, 4.42, 4.36, 4.26, 3.85, 4.47 and 3.77, respectively, which were far greater than 2.0, and their high efflux ratios were decreased significantly in the presence of 10 µM verapamil. In addition, the effect of verapamil on the uptake of ginsenosides had been investigated, and the results are shown in Figure 3(D). The uptake of digoxin, ginsenoside Rb2, Rc, Rg2, Rg3, Rd and Rb1 was found can be significantly enhanced by verapamil.

In summary, the results above indicated that the ginsenoside Rb2, Rc, Rg2, Rg3, Rd and Rb1 were moderate P-gp substrates, and ginsenosides Rh1, F1, Re, and Rg1 almost could not be transported by P-gp.

### The effects of SLE on ginsenosides transport and uptake

According to our previous study [22], SLE was a strong P-gp inhibitor, and could significantly decrease the ERs of P-gp substrates (digoxin and vincrisine) in L-MDR1 and Caco-2 cells. Herein, the effects of SLE on the transport and uptake of ginsenoside Rb2, Rc, Rg2, Rg3, Rd and Rb1 would be investigated since these ginsenosides were been proven as moderate P-gp substrates based on the experiments above.

#### Inhibition of ginsenosides transport by SLE in cultured LLC-PK1 and L-MDR1 cells

**Figure 4:**
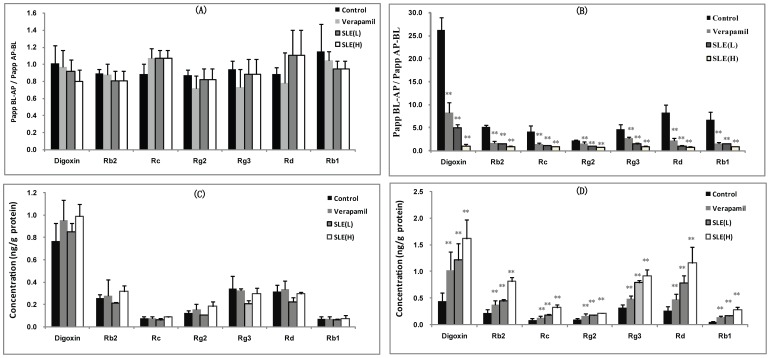
The effect of SLE on the uptake and transport of digoxin and ginsenosides in LLC-PK1 and L-MDR1 cell. (A) The transport of digoxin and ginsenosides on LLC-PK1 cell in the absence and presence of SLE at 2.0 µg/mL (L) and 10.0 µg/mL (H). (B) The transport of digoxin and ginsenosides on L-MDR1 cell in the absence and presence of SLE at 2.0 µg/mL (L) and 10.0 µg/mL (H). (C) The concentration of digoxin and ginsenosides in LLC-PK1 cell in the absence and presence of SLE at 2.0 µg/mL (L) and 10.0 µg/mL (H). (D) The concentration of digoxin and ginsenosides in L-MDR1 cell in the absence and presence of SLE at 2.0 µg/mL (L) and 10.0 µg/mL (H).

Herein, we investigated the effect of SLE on the transport of ginsenosides to explore the compatibility rule between ginsenosides and SLE in *Shengmai-san* based on P-gp. Figure 4(A) illustrates the absorptive (AP→BL) and secretory (BL→AP) transport of ginsenoside Rb2, Rc, Rg2, Rg3, Rd, Rb1 and digoxin on LLC-PK1 cells (lacking P-gp), in the absence and presence of SLE (2.0 and 10.0 µg/mL) as P-gp inhibitor. No transport difference was observed when SLE was added. It was mean that the efflux of ginsenoside Rb2, Rc, Rg2, Rg3, Rd, Rb1 and digoxin across LLC-PK1 cell monolayer in the absorptive (AP→BL) and secretory (BL→AP) directions and the corresponding *P*_app_ values were not affected by SLE. On the other hand, we also compared the transport of ginsenoside Rb2, Rc, Rg2, Rg3, Rd, Rb1 and digoxin on L-MDR1 cells, in the absence and presence of SLE (2.0 and 10.0 µg/mL) as P-gp inhibitor (shown in Figure 4(B)). Obviously, the ERs of ginsenoside Rb2, Rc, Rg2, Rg3, Rd, Rb1 and digoxin could be significantly decreased by SLE.

#### The Effect of SLE on the uptake of ginsenosides in cultured LLC-PK1 and L-MDR1 cells

Herein, we examined the effect of SLE on the uptake of ginsenosides on LLC-PK1 cells and the derived cell line L-MDR1. As shown in Figure 4(C), intracellular accumulation of ginsenoside Rb2, Rc, Rg2, Rg3, Rd, Rb1 and digoxin on LLC-PK1 cells had no significant difference in the absence and presence of SLE. In contrast, SLE concentration dependently increased intracellular concentrations of ginsenoside Rb2, Rc, Rg2, Rg3, Rd and Rb1 in L-MDR1 cells. When 2.0 µg/mL SLE was added, the intracellular concentrations of ginsenoside Rb2, Rc, Rg2, Rg3, Rd, Rb1 and digoxin in L-MDR1 cells were increased by 2.0-, 1.9-, 1.7-, 2.5-, 2.9, 2.9 and 2.7- fold. The intracellular concentrations of ginsenoside Rb2, Rc, Rg2, Rg3, Rd, Rb1 and digoxin in L-MDR1 cells were increased by 3.7-, 3.5-, 2.1-, 2.9-, 4.3, 5.1-, and 3.6- fold with the presence of 10.0 µg/mL SLE (Figure 4(D)).

#### Inhibition of ginsenoside Rb2, Rc, Rg2, Rg3, Rd and Rb1 transport across Caco-2 monolayer by SLE

**Figure 5:**
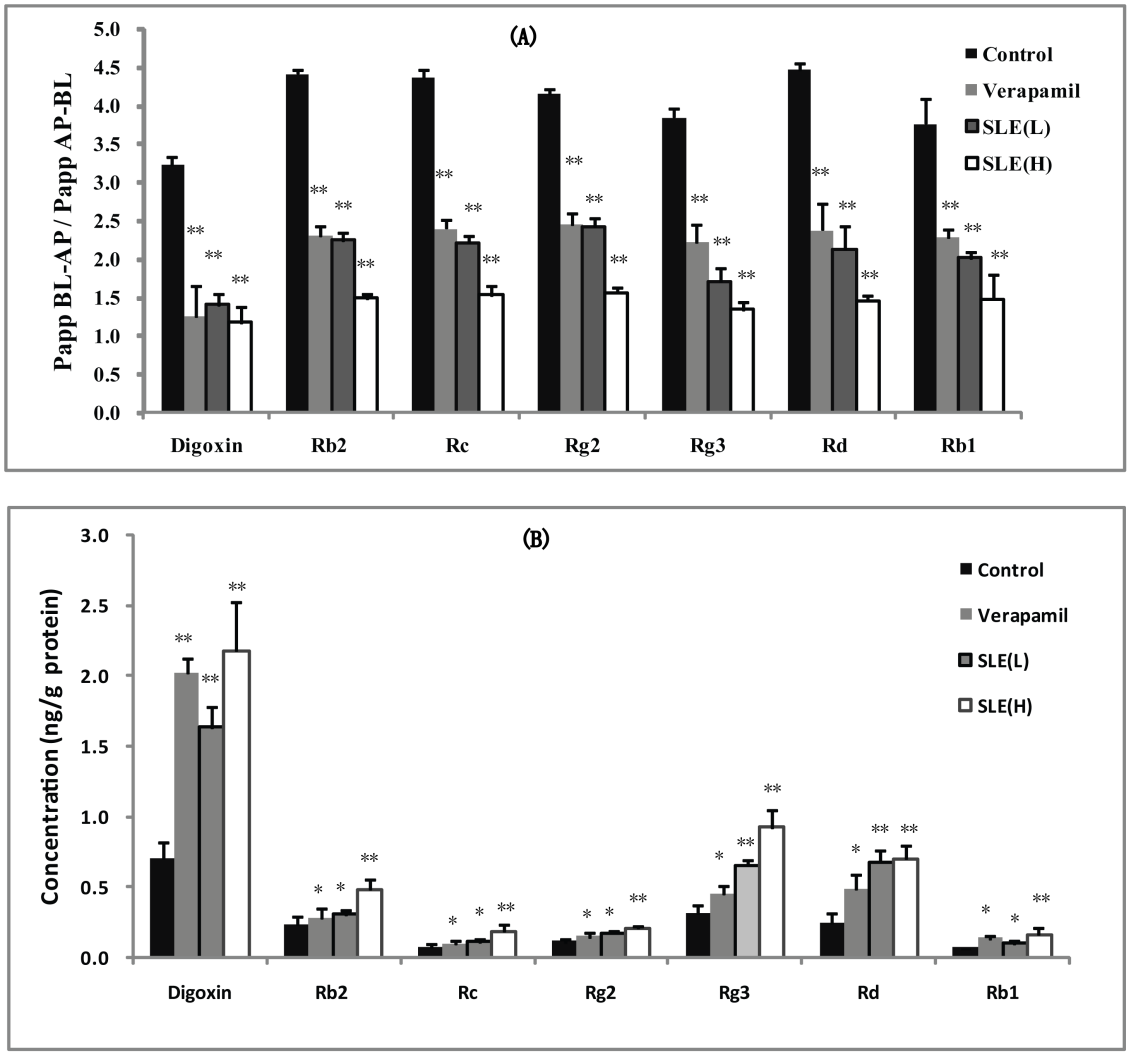
The effect of SLE on the transport and uptake of digoxin and ginsenosides in Caco-2 cell. (A) The transport of digoxin and ginsenosides on Caco-2 cell in the absence and presence of SLE at 2.0 µg/mL (L) and 10.0 µg/mL (H). (B) The uptake of digoxin and ginsenosides in Caco-2 cell in the absence and presence of SLE at 2.0 µg/mL (L) and 10.0 µg/mL (H).

The ability of SLE to affect the transport of ginsenoside Rb2, Rc, Rg2, Rg3, Rd and Rb1 was further confirmed in Caco-2 cells. Ginsenoside Rb2, Rc, Rg2, Rg3, Rd and Rb1 exhibited highly polarized transport across Caco-2 cell monolayer with marked efflux ratio values at 4.42, 4.36, 4.26, 3.85, 4.47 and 3.77, respectively, which were far greater than 2.0, and the presence of SLE could concentration dependently decrease the transport capability of ginsenoside Rb2, Rc, Rg2, Rg3, Rd and Rb1 across Caco-2 monolayer in the BL-AP direction, which correspondingly led to the decrease in efflux ratios (As shown in Figure 5(A)).

#### Enhancing uptake of ginsenoside Rb2, Rc, Rg2, Rg3, Rd and Rb1 by SLE in Caco-2 cell

The effect of SLE on the uptake of ginsenoside Rb2, Rc, Rg2, Rg3, Rd and Rb1 had been investigated and the results are shown in Figure 5(B)). SLE could concentration dependently increase intracellular concentrations of ginsenoside Rb2, Rc, Rg2, Rg3, Rd and Rb1 in Caco-2 cells when 2.0 and 10.0 µg/mL of SLE were loaded (*P<0.01*).

#### Single-dose SLE increased plasma concentrations of ginsenosides in rats

**Figure 6:**
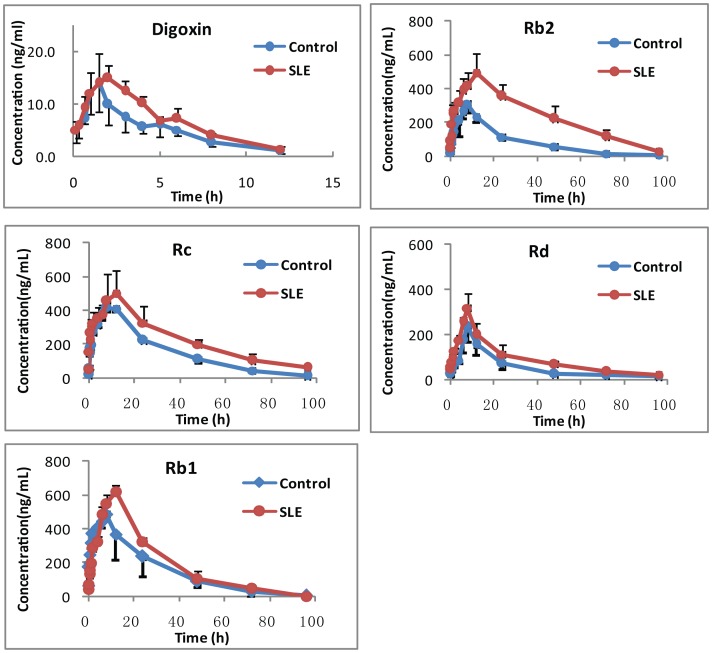
The plasma concentration-time profiles of digoxin and ginsenoside Rb1, Rb2, Rc and Rd after single-dose of vehicle / SLE and GE.

In this process, the effect of single-dose SLE on the plasma pharmacokinetics of ginsenosides in rats was investigated. The plasma concentration of ginsenosides after intragastric administration of GE at the dose of 150 mg/kg or in combination with SLE was determined by LC/MS. As a result, ginsenoside Rb1, Rb2, Rc and Rd were the main components in rat plasma, and the exposure of ginsenoside Rb1, Rb2, Rc and Rd was much higher than other components. The plasma concentration-time profiles of ginsenoside Rb1, Rb2, Rc and Rd are shown in Figure 6 and their pharmacokinetic parameters are shown in Table 1. Obviously, coadministration of SLE could evidently increase the AUC_0-∞_ of ginsenoside Rb2 for 2.18-fold, from 10551.63±1728.81 ng.h/mL in the vehicle treated group to 23198.61±5566.23 ng.h/mL in the SLE-treated group (*P<0.01*). For ginsenoside Rc and Rd, similarly, coadministration of SLE could increase the AUC for 1.49-fold and 1.86-fold (*P<0.05*). For ginsenoside Rb1, coadministration of SLE could increase the AUC for 1.29-fold, but no statistical difference was found between the two groups after carrying *t* test (*P>0.05*). Besides, maximum plasma concentrations (*C*_max_) for Rb1 and Rb2 were also significantly enhanced by SLE treatment (*P<0.05*), and *C*_max_ of ginsenoside Rc and Rd was not dramatically changed after SLE treatment. At the same time, the time of maximum plasma concentration (*T*_max_) and half-life time (*t_1/2_*) were not altered after coadministration of SLE. As a positive control, the effect of single-dose SLE on the pharmacokinetics of digoxin in rats was investigated. The plasma concentration-time profiles when digoxin was single administered alone or in combination with SLE at 500 mg/kg are shown in Fig. 6 and the pharmacokinetic parameters are list in Table 1. Obviously, coadministration of SLE could significantly increase the AUC_0-τ_ of digoxin 1.25-fold, from 61.80±13.37 ng.h/mL in the vehicle treated group to 84.38±7.25 ng.h/mL in the SLE-treated group.

**Table 1:** The pharmacokinetic parameters of digoxin and ginsenoside Rb1, Rb2, Rc and Rd after single ig SLE and 150/kg GE in rats (n = 5).

	Ginsenosides	t_1/2_ (h)	T_max_ (h)	C_max_ (ng/mL)	AUC_all_ (ng.h/mL)	AUC_INF_obs_ (ng.h/mL)	Cl__F_obs_ (L/h)	MRT_INF_obs_ (h)
Control	Digoxin	3.08±1.23	1.28±0.36	15.04±5.07	61.80±13.37	67.51±13.43	7.66±1.53	5.15±1.09
	Rb1	13.34±2.43	7.50±1.00	491.40±61.38	14256±2378	14394±2469	0.007±0.001	23.10±4.31
	Rb2	19.42±2.87	7.00±2.00	330.46±70.86	10224±1706	10551±1728	0.012±0.003	27.91±4.20
	Rc	16.90±2.14	9.00±2.00	429.99±16.66	14523±879	14877±876	0.007±0.000	28.30±1.70
	Rd	75.42±42.74	7.50±1.00	236.51±79.05	5347±1405	7409±2177	0.014±0.003	21.63±48.98
SLE	Digoxin	2.98±0.36	1.58±0.38	17.46±2.26	84.38±7.25**	90.98±8.39**	5.53±0.48**	5.06±0.42
	Rb1	11.34±2.74	11.00±2.00	650.56±126.56	18457±5779	18511±5733	0.006±0.002	23.53±2.68
	Rb2	19.03±3.84	9.50±3.00	540.94±45.03	22310±5273**	23198±5566**	0.004±0.001	25.17±3.63
	Rc	17.34±4.54	9.50±3.00	514.89±142.09	21642±4411*	24127±3845*	0.004±0.001	23.61±8.09
	Rd	21.83±6.78	7.50±1.00	227.47±57.82	7962±2002*	9045±2203*	0.011±0.003	25.09±7.45

In control group: the rats were intragastrically administrated a single dose of vehicle (0.5% sodium CMC), 2 h later, digoxin (0.5 mg/kg) / GE (120 mg/kg) was given to the rats by intragastric administration. In SLE group: the rats were administrated a single dose of SLE intragastrically at 500 mg/kg suspended in 0.5% sodium CMC. 2 h later, digoxin (0.5 mg/kg)/GE (120 mg/kg) was given to the rats by intragastric administration.

*:P<0.05 & **:P<0.01.

#### Long-term treatment with SLE increased plasma concentrations of ginsenosides in rats

**Figure 7:**
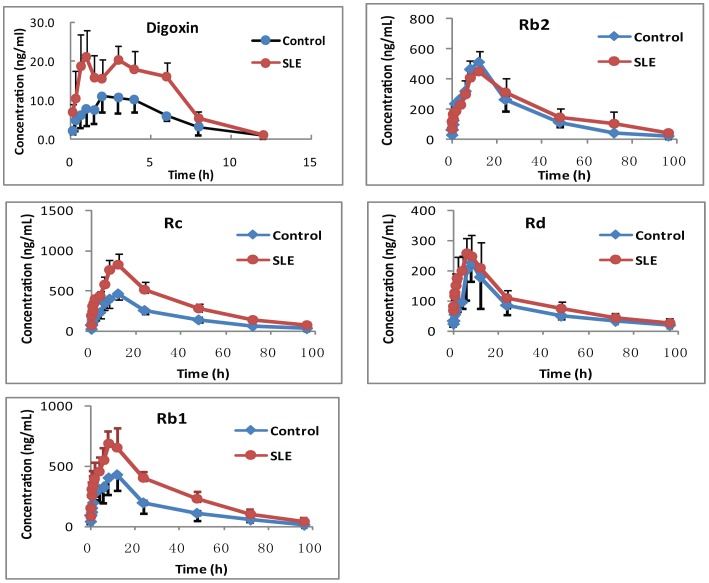
The plasma concentration-time profiles of digoxin and ginsenoside Rb1, Rb2, Rc and Rd after multi-dose of vehicle / SLE and GE.

In order to investigate the effect of long-term treatment with SLE on the pharmacokinetics of ginsenosides, 5 rats were given a dose of SLE intragastrically at 500 mg/kg suspended in 0.5% sodium CMC once a day for 10 continuous days, whereas the other 5 rats received the vehicle (0.5% sodium CMC), serving as the control groups. On the 11^th^ day, GE was administered to rats at a single dose of 150 mg/kg by gavage. As shown in Figure 7, 10 continuous days administration of SLE could increase the AUC_0-∞_ of ginsenoside Rb2 for 1.33-fold, from 22557.12±6121.56 ng.h/mL in the vehicle treated group to 16600.84±3511.26 ng.h/mL in the SLE-treated group, but no statistical difference was found between the two groups after carrying *t* test (P>0.05). For ginsenoside Rc and Rd, similarly, coadministration of SLE could increase the AUC for 1.16-fold and 1.60-fold. Besides, 10 continuous days administration of SLE could increase the AUC_0-∞_ of ginsenoside Rb1 1.82-fold, from 14324.65±5178.69 ng.h/mL in the vehicle treated group to 27017.32±2381.53 ng.h/mL in the SLE-treated group (*P<0.01*). The pharmacokinetic parameters of of ginsenoside Rb1, Rb2, Rc and Rd in rats are shown in Table 2. Besides, the effect of multi-dose SLE on the pharmacokinetics of digoxin (a positive drug) in rats was investigated. 10 continuous days administration of SLE could increase the AUC_0-τ_ of digoxin about 2-fold, from 68.30±25.96 ng.h/mL in the vehicle treated group to 123.24±52.78 ng.h/mL in the SLE-treated group (*P<0.05*).

**Table 2:** The pharmacokinetic parameters of digoxin and ginsenoside Rb1, Rb2, Rc and Rd after multiple ig SLE and 150/kg GE in rats (n = 5).

	Ginsenosides	t_1/2_ (h)	T_max_ (h)	C_max_ (ng/mL)	AUC_all_ (ng.h/mL)	AUC_INF_obs_ (ng.h/mL)	Cl__F_obs_ (L/h)	MRT_INF_obs_ (h)
Control	Digoxin	2.99±2.32	2.33±1.03	11.75±4.22	68.30±25.96	76.14±30.04	7.48±3.09	5.65±2.85
	Rb1	17.24±2.16	10.00±4.47	430.32±135.11	14066±5067	14324±5178	0.0079±0.0035	28.09±4.81
	Rb2	20.15±3.66	12.00±0.00	512.33±77.75	15937±3310	16600±3511	0.0062±0.0013	29.50±2.61
	Rc	20.81±9.64	11.20±1.79	475.56±64.04	12569 ±1821	14462 ±2343	0.0143±0.0035	22.35±9.98
	Rd	20.81±9.64	8.00±2.45	261.64±103.87	6569±1821	7462±2343	0.0143±0.0035	22.35±9.98
SLE	Digoxin	2.19±0.87	3.28±1.73	22.94±13.67*	123.24±52.78*	126.79±52.07*	4.49±1.75*	4.78±0.91
	Rb1	19.31±8.29	9.60±2.19	735.89±142.95	25644±1305**	27017±2381**	0.0037±0.0003	33.50±7.91
	Rb2	24.28±8.42	10.00±2.83	585.87±95.24	21261±6522**	22557±6121**	0.0047±0.0011	26.51±6.58
	Rc	26.75±3.08	11.20±1.79	422.52±86.49	14641±2687	16729±8560	0.0028±0.0002	40.03±4.04
	Rd	17.97±75.93	8.00±2.45	264.68±49.43	7641±2687	11729±8560*	0.0125±0.0084	26.32±90.58

In control group: rats were given the vehicle (0.5% sodium CMC) once a day for 10 continuous days. On the 11^th^ day, digoxin (0.5 mg/kg) / GE (120 mg/kg) was administered intragastrically to rats. In SLE+GE group: rats were given a dose of SLE intragastrically at 500 mg/kg suspended in 0.5% sodium CMC once a day for 10 continuous days. On the 11^th^ day, digoxin (0.5 mg/kg) / GE (120 mg/kg) was administered intragastrically to rats.

*:P<0.05 & **:P<0.01.

## Discussion and Conclusions

Over 1,000 Chinese herb formulae have been used in China since hundreds of years and their modern preparations are also widely applied in clinic [7]. In the tide of the modernization of Chinese medicine, combined application of TCM can achieve a synergistic interaction capable of yielding more distinct effect at lower doses than that produced by single Chinese herbal medicine [33]. *Shengmai-san* has protective effects against oxidative damages in the cells or tissues of the cardiovascular and nervous systems, and play an important role on treatment of symptoms related to cardiovascular diseases such as heart failure, stroke, shock, *etc* [34]. *Schisandra Chinensis* in *Shengmai-san* formulae, a strong P-gp inhibitor, was used as an adjuvant drug to improve the pharmacological activity of monarch drug (*Panax Ginseng*). In order to investigate the mechanism of enhancing the pharmacological activity produced by *Schisandra Chinensis*, the influence of SLE on the pharmacokinetics of the ginsenosides was systematically investigated on *in vitro* and *in vivo* models based on P-glycoprotein.

Firstly, the effect of P-gp inhibitor (verapamil) on the transport of ginsenosides in LLC-PK1 cells and the derived cell line L-MDR1 were compared to investigate which ginsenosides were P-gp substrates. As a result, ERs of ginsenosides across LLC-PK1 cell monolayers and the corresponding *P*_app_ values were not affected by verapamil, while the use of verapamil caused a significantly decrease on ERs of ginsenosides (Rb2, Rc, Rg2, Rg3, Rd and Rb1) in L-MDR1 cells. Then, the uptake of ginsenoside Rb2, Rc, Rg2, Rg3, Rd and Rb1 in L-MDR1 cells was found be greatly increased by verapamil, while the uptake of ginsenoside Rh1, F1, Re, and Rg1 in LLC-PK1 cell was found almost not affected by verapamil. According to the results above, the ginsenoside Rb2, Rc, Rg2, Rg3, Rd and Rb1 were identified as P-gp substrates. In 2006, Caco-2 cell monolayer was used as an *in vitro* model to reveal the transport mechanism of Rb1 across the intestinal mucosa [21]. Ginsenoside Rb1 was found not be transported by P-gp, which was contradictory to our research. The main possible reason was that the expression and activity of P-gp on Caco-2 cell were not high enough to confirm whether Rb1 was a moderate P-gp substrate or not. Besides, existence of other transporters and metabolic enzymes on Caco-2 cell may be another reason.

Based on the research above, it seems that an important breakthrough on the compatibility of *Ginseng* and *Schisandra Chinensis* in *Shengmai-san* was found, and SLE may enhance the exposure of ginsenosides through inhibiting the activity and expression of P-gp. In order to confirm the views above, the effect of SLE on the pharmacokinetics of ginsenosides was systematically investigated based on both *in vitro* and *in vivo* models. As we infer, the efflux ratios of ginsenoside Rb2, Rc, Rg2, Rg3, Rd and Rb1 in L-MDR1 cells could be significantly decreased by SLE, and the transport of ginsenoside Rh1, F1, Re, and Rg1 was found almost not be affected by SLE. Besides, the intracellular concentration of ginsenoside Rb2, Rc, Rg2, Rg3, Rd and Rb1 on L-MDR1 cells was greatly increased with the presence of SLE in L-MDR1 cells. In contrast, the transport and uptake could not be altered by SLE in the LLC-PK1 cell. Likewise, the effects of SLE on the transport and uptake of ginsenosides were also investigated in Caco-2 cell which was always used to assess bioavailability for several years. As a result, SLE could concentration dependently decrease the efflux ratios and increase the uptake of ginsenoside Rb2, Rc, Rg2, Rg3, Rd and Rb1 in Caco-2 cell. In the *in vivo* model, single administration of SLE was found can increase the AUC of ginsenosides (Rb1, Rb2, Rc and Rd), and the exposure of ginsenoside Rb1, Rb2, Rc and Rd could also be significantly enhanced by multiple administration (for 10 continuous days) of SLE.

Overall, our results suggested that the exposure of ginsenoside Rb2, Rc, Rg2, Rg3, Rd and Rb1 could be enhanced by SLE, and the compatibility law of *Ginseng* and *Schisandra Chinensis* in *Shengmai-san* could be explained based on P-gp regulation to some extent.

## Supporting Information

Figure S1 **The structures of ginsenoside Rh1, F1, Rb2, Rc, Rg2, Rg3, Re, Rd, Rb1, Rg1 and digoxin (internal standard).**
(TIF)

Figure S2 **The chromatography of ginsenoside Rh1, F1, Rb2, Rc, Rg2, Rg3, Re, Rd, Rb1, Rg1 and digoxin (internal standard).**
(TIF)
