# Supplementary figures and images for: Pharmacokinetic Compatibility of Ginsenosides and Schisandra Lignans in Shengmai-san: From the Perspective of P-Glycoprotein
Source: PLoS One. 2014 Jun 12;9(6):e98717. doi: 10.1371/journal.pone.0098717 (PMC4055595; doi:10.1371/journal.pone.0098717)

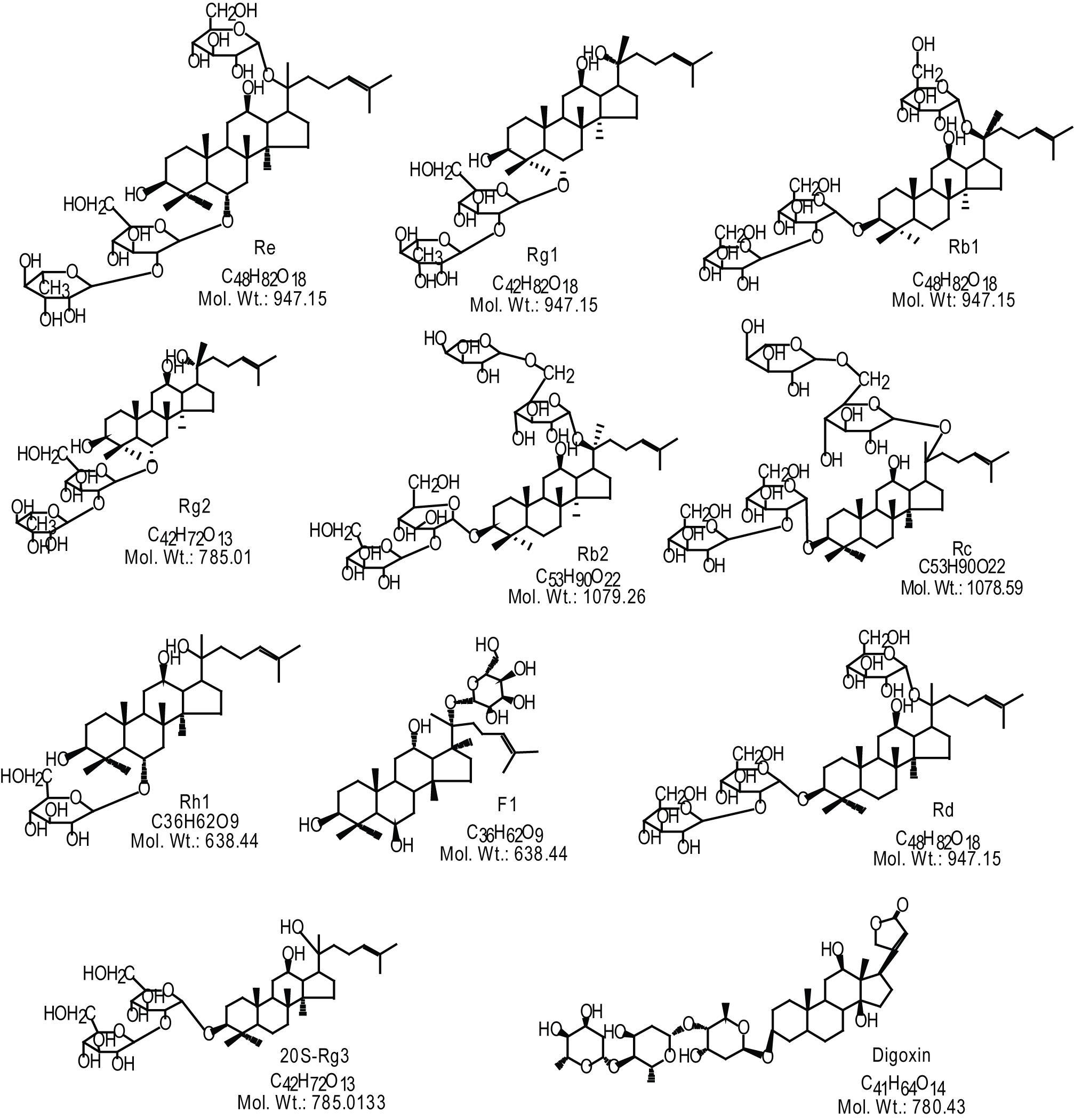

Supplement: Figure S1 — The structures of ginsenoside Rh1, F1, Rb2, Rc, Rg2, Rg3, Re, Rd, Rb1, Rg1 and digoxin (internal standard). (TIF) [file pone.0098717.s001.tif]

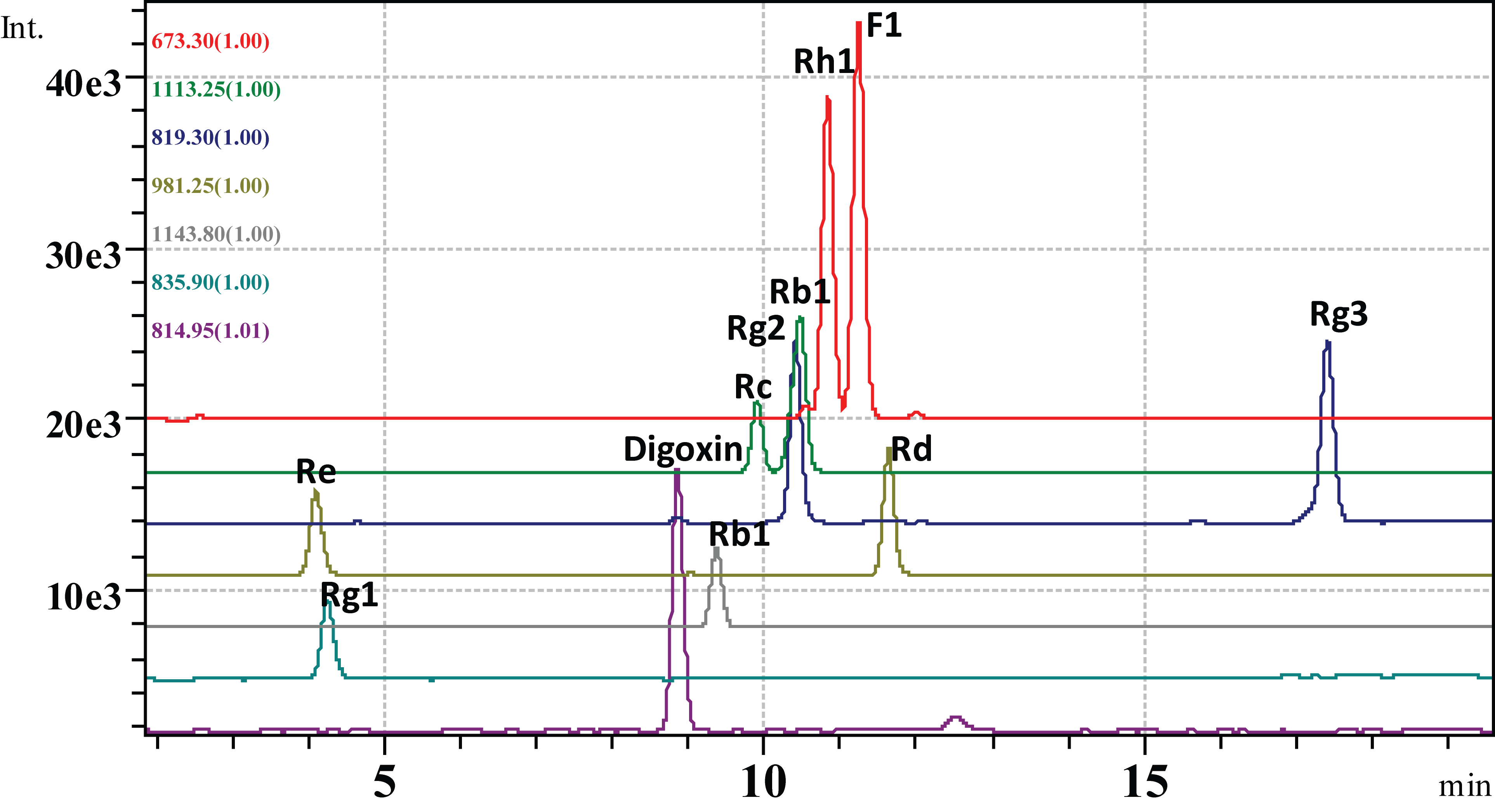

Supplement: Figure S2 — The chromatography of ginsenoside Rh1, F1, Rb2, Rc, Rg2, Rg3, Re, Rd, Rb1, Rg1 and digoxin (internal standard). (TIF) [file pone.0098717.s002.tif]
